# Diverticule congénital de la vessie chez un enfant de 13 ans diabétique en sepsis à point de départ urinaire

**DOI:** 10.11604/pamj.2013.14.56.2357

**Published:** 2013-02-11

**Authors:** Zine El Abidine Benali, Houssam Ahmaidi

**Affiliations:** 1 Service d'anesthésie - réanimation, Hôpital Provincial Eddarak, Berkane, Maroc

**Keywords:** Diverticule congénital, urine, vessie, diabète, congenital diverticulum, urine, bladder, diabetes

## Image en médicine

Les diverticules congénitaux dans la population pédiatrique sont très rares et les données actuelles sont insuffisantes pour fournir la différence de fréquence dans les deux sexes .Par définition, C'est une hernie de la couche interne de la paroi de la vessie, La déficience congénitale ou une faiblesse dans la gaine aponévrotique de Waldeyer a été considérée comme une cause. Les diverticules congénitaux ont tendance à être solitaires et se trouvent à la jonction du trigone de la vessie et du détrusor. Cette localisation anatomique, près de l'insertion de l'uretère jusqu'à la vessie, est de considération importante parce que les diverticules de grande taille peuvent empiéter sur ou déformer les orifices urétéraux. Par conséquent, procéder à l'exérèse chirurgicale de ces diverticules avec soin pour éviter de blesser l'uretère. Nous rapportons le cas d'un patient âgé de 13 ans, diabétique insulinodépendant hospitalisé en réanimation pour un sepsis à point de départ urinaire, l’échographie au lit du malade a montré une image anéchogène parallèle à la vessie, le doppler couleur avec pression dosée sur une vessie pleine montre le passage des urines à travers le diverticule. Après un traitement médical orienté par l'antibiogramme, l'enfant est opéré sous rachianesthésie 1 mois après sans séquelles.

**Figure 1:**
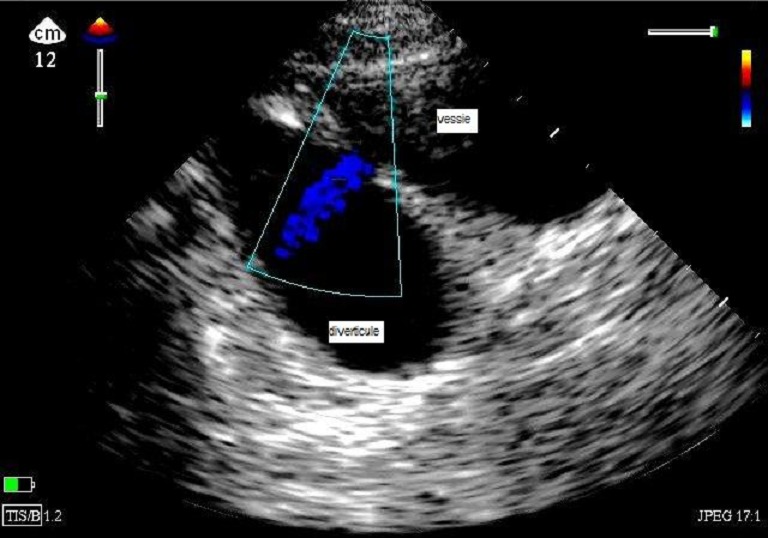
Image échographique mode 2 D avec doppler couleur montrant le passage des urines à travers le diverticule après pression dosée sur une vessie pleine chez un enfant de 13 ans

